# Temporal Dynamics of Vaccine Uptake: Perceptual and Social Drivers of Adoption Speed Across Innovation Diffusion Curve

**DOI:** 10.3390/microorganisms14051049

**Published:** 2026-05-07

**Authors:** Rungting Tu, Cheryl Lin, G. Natasha Santoso, Wendy E. Braund, Ann M. Reed, Pikuei Tu

**Affiliations:** 1 Department of Business Administration, Tunghai University, Taichung 407, Taiwan; 2 Policy and Organizational Management Program, Duke University, Durham, NC 27708, USA; c.lin@duke.edu (C.L.);; 3 Independent Researcher, Fayetteville, NY 13066, USA; 4 School of Medicine, Duke University, Durham, NC 27708, USA

**Keywords:** public health, SARS-CoV-2, vaccine hesitancy, risk perception, innovation adoption, social influence, norm, health behavior, decision-making, mediator

## Abstract

The effectiveness of infection prevention depends on not only uptake but also the timing of adoption. Vaccination studies typically treat uptake as binary, overlooking *when* while investigating *why* individuals get vaccinated. Using the novel mRNA COVID-19 vaccines as a case study, the influences of risk perceptions and social norms on vaccination timing were examined through an Innovation Diffusion framework. An online survey was conducted in November 2021 to assess vaccination behaviors, attitudes, and peer expectations of 1710 U.S. residents (51.64% females, 31.23% minorities, with a relatively balanced distribution across age and income brackets). Participants were classified by vaccination timing and intentions as early adopters, early majority, late majority, or laggards for comparative analyses. One year after vaccine rollout, 64.3% had received at least one dose; 20.1% reported no intention to vaccinate, and this resistance persisted through May 2023 when the pandemic ended. Vaccine confidence and prior behavior (e.g., influenza vaccination) demonstrated strong gradients across adoption timing. Earlier uptake was associated with higher perceived vaccine importance, infection risk, and peer uptake, whereas age and education effects diminished over time. Perceived illness severity and disease knowledge showed inconsistent influences. Later adopters anticipated higher post-vaccination infection risk and greater peer non-vaccination, reinforcing hesitancy. Social norms (but not risk perception) mediated the relationship between confidence and timing; earlier adoption further predicted booster acceptance. These findings highlight the importance of trust, correcting efficacy misperceptions, and leveraging positive peer norms to promote timely vaccination and inform strategies for other infectious diseases.

## 1. Introduction

The effectiveness of a scientific invention or medical breakthrough, be it an infection preventive procedure or novel treatment, largely depends on whether the intended population is willing and able to accept the new measure and obtain it in time. Despite the availability of numerous safe and effective vaccines, many individuals delay or refuse vaccination—a phenomenon known as vaccine hesitancy that impedes progress toward herd immunity against infectious diseases [1,2,3]. This challenge is especially pronounced and concerning during regional or global outbreaks, as seen during the COVID-19 pandemic, when prompt and mass uptake was essential to curb transmission and support economic recovery [4].

The Centers for Disease Control and Prevention’s (CDC) Immunization Services Division distributed the COVID-19 vaccine to each of the CDC’s 64 jurisdictions (50 states, the District of Columbia, five cities and eight territories). Census data and vaccine availability projections were used to determine jurisdictional vaccine allocations; each jurisdiction developed its own COVID-19 allocation framework using general guidance from the CDC and available data on critical and vulnerable populations. Although the plans were provided to the CDC, state and local health departments determined how many doses to distribute to the enrolled providers in their jurisdiction [5,6].

Even when the new mRNA vaccine was provided free to the public, with costs covered by the U.S. government, only 61.43% of the American population were fully vaccinated by the end of 2021, one year after the launch of the vaccine (compared to 74.91% in France, 69.54% in the United Kingdom (UK), and 79.77% in Japan) [7]. Likewise, for the seasonal flu shot, recommended for people six months and older but not required (except for certain professions like military and healthcare) and fully covered by both public and private insurance in the U.S., the vaccination rates have hovered around 41–63%, varying by state and declining since the pandemic [8,9].

To understand vaccine decision-making, researchers have drawn on behavioral models such as the Health Belief Model (HBM) [10,11,12], Theory of Planned Behavior (TPB) [12,13,14], and Precaution Adoption Process Model (PAPM) [15,16]. The PAPM emphasizes how knowledge, provider reassurance, and social norms influence Human Papillomavirus (HPV) vaccine acceptance [16]. Similarly, the HBM links acceptance to perceived efficacy, risk, and provider recommendation, and identifies barriers such as cost and concerns that vaccinating against a common sexually transmitted infection (STI) like HPV may be seen as implicitly promoting adolescent sexual activity [12,17]. Though these models provide a cognitive, individual-level explanation of behavior, they often investigate vaccination as a dichotomy and fall short of accounting for or predicting the timing or speed of adoption and the ratio of unprotected population across broader social systems at a particular phase. These dynamics could determine the length of an outbreak and the risk of it turning into an epidemic.

The Diffusion of Innovations (DOI) theory, developed by Everett Rogers with origins in sociology and communication [18], addresses this gap by examining how new ideas and technologies are spread within populations. It categorizes individuals into five groups based on adoption timing: innovators, early adopters, early majority, late majority, and laggards [19]. The categorization reflects differences in response to new information, uncertainty, and social influence. DOI also identifies five innovation characteristics that shape adoption: relative advantage, compatibility, complexity, trialability, and observability [19]. The theory has been applied globally in healthcare [20,21], technology (e.g., e-health [22] and patient portals [23]), and public policy [24,25]. For instance, the DOI theory was used to successfully predict healthcare workers’ readiness to adopt E-Health platforms in a Mauritian hospital setting, with early adopters serving as key leaders in driving diffusion across adopter categories [22]. Preventive innovations like vaccines often diffuse slowly, as their benefits are less immediately visible and their value may be difficult to comprehend or assess [26].

In influenza and HPV vaccination studies, DOI has helped demonstrate how factors like alignment with individual beliefs, access, and trusted messengers (e.g., school nurses [27]) motivated uptake over time [27,28,29]. However, peer-to-peer influence, despite its central role in the theory, has largely been overlooked. Many studies have focused on trusted sources like healthcare providers or pastors [30,31], but people also take cues from friends, coworkers, and others in their social circles [32]. Recognizing the potential sway of these personal networks, or “observability” as the DOI describes, can bring deeper insight into the social drivers of vaccination behavior. Further, many studies inquired about intentions or hypothetical responses rather than the action of actual uptake [16,33,34,35,36], which may not fully represent the behavior or capture changes in perceptions and contextual factors at the time of decision.

The COVID-19 vaccine offers a unique and compelling case for applying DOI theory in studying real-time adoption behavior: it was the first mRNA vaccine with FDA approval [37], beneficial to populations worldwide yet unfamiliar to the public. Some obtained the vaccine as soon as it became available, and others stalled for months or longer—or never accepted it [38,39]—despite widespread access, vast promotion, and amidst increasing confirmed cases nationally and worldwide [40]. In September 2020, two regional polls in North Carolina and Iowa, respectively, found 20.6% and 28.0% respondents said they would take the shot as soon as they could, while 51.0% and 45% chose to wait until others have taken it [41,42]. DOI’s core premise that adoption depends on perceived risk, benefit, and trust provides a temporal framework to examine the progression of uptake. A few COVID-19 studies referenced DOI, but not all applied the full model in comparing the timing of adoption or considered the influence of social perception. For instance, a study in Cacao, China, examined COVID-19 vaccination timing among tourism workers, integrating DOI with TPB and finding that vaccine attitude, perceived behavioral control, and prior influenza vaccination history predicted earlier uptake [43]. While this work demonstrates the value of a timing-based, DOI-informed approach in a non-Western context, it was limited to a single high-risk occupational group and did not explore peer-to-peer social influence.

Building on prior research, the objective of this study was to investigate the uptake progression via the DOI framework with a nationally representative sample in assessing not just who obtained vaccination but *when* and *why*. This approach aimed to overcome the shortfall of binary classification in most studies contrasting vaccinated vs. unvaccinated individuals, which oversimplifies the complex interplay of social, psychological, and contextual factors [1,16]. Specifically, actual adoption timing (or intention, for those not yet vaccinated) and the factors shaping it are analyzed, including common behavioral drivers like perceived infection risk and past vaccination behavior (e.g., annual flu shot) [39,44,45], as well as how self-perceived knowledge, external conditions, and peer influence affect vaccine decisions across adopter categories. The findings could help inform healthcare providers and policymakers in understanding and applying the differing effects of relevant factors that motivate vaccination adoption, encourage early action, or delay uptake.

## 2. Methods

### 2.1. Study Design and Participants

An online survey was conducted in November-December 2021, approximately one year after the launch of the first COVID-19 vaccine, to assess participants’ and their peers’ vaccination status, attitudes and experience relating to the COVID-19 pandemic and vaccine, along with demographic information (age, gender, race, education, and household income). To minimize selection bias, a stratified quota sampling approach was employed with the support of a professional panel company to strengthen the representativeness of the sample. Potential participants were only notified about the opening of the survey and not informed of the subject matter to avoid self-selection bias that may lead to oversampling people who had particularly strong opinions about the pandemic or vaccine. Unique to COVID-19 vaccination, most people remembered the month of their first dose at the time of the survey because of the widespread attention to the initial eligibility prioritization, the need to make and often wait for an appointment, and the required interval to receive the second dose or a booster (or they could check the date on their vaccination card), which reinforced memory.

The selection of indicators was based on theory, the literature review, and study objectives. The questionnaire was reviewed and advised by an expert panel and pilot tested with a small, diverse group resembling the intended sample before fielding. Because COVID-19 vaccines became available through emergency authorization in December 2020 for people aged 16 and older, the target population included U.S. residents aged 16+ (approximately 275 million) [46]. With a goal of achieving a 98% confidence level and 3% margin of error, the sample size was estimated to be at least 1509 [47]. Participants were recruited through a panel company to reach a nationally representative sample. The study protocol was approved by Duke University’s Institutional Review Board. Each participant indicated their consent by clicking the “next” button at the end of the informed consent webpage to start the survey.

### 2.2. Measures

#### 2.2.1. Vaccination Status and DOI Group Categorization

The primary outcome variable was vaccination timing. Participants reported their COVID-19 vaccine status with six answer options: 1—No, I do not plan to get it; 2—I am not sure whether to get it; 3—I will (or plan to) get it; 4—I received one dose of the Pfizer or Moderna vaccine, but I am not sure if I want to get the second dose; 5—I received one dose and will get the second dose soon; and 6—I am fully vaccinated. Those vaccinated (responded 4–6) then reported the month of their first dose.

Following Rogers’ DOI theory [18], participants were initially grouped into five categories—innovators, early adopters, early majority, late majority, and laggards—based on the month (or absence) of their first COVID-19 vaccine dose. Due to the small number of innovators, as well as the initial vaccine shortage and limited eligibility that might have delayed some eager individuals from obtaining the vaccine, this group was merged with early adopters, resulting in four categories for statistical analysis.

Participants vaccinated between December 2020 and March 2021 were classified as *early adopters* (23%), April–July 2021 as *early majority* (30.6%), after July 2021 or those expressing intent or uncertainty about vaccination as *late majority* (26.4%), and those with no intention to get vaccinated as *laggards* (20.1%). This distribution aligns with CDC-reported national trends: by December 2021, 68.6% of the U.S. population had received at least one dose, rising to 78.43% by December 2022 [7]. The proportion of laggards in this study (20.1%) closely mirrors the ~20% who remained unvaccinated at the end of the pandemic (May 2023) [7].

#### 2.2.2. Risk Perceptions, Attitudes, and Knowledge

Independent variables were selected based on prior literature on vaccine uptake and hesitancy. Risk-related measures included perceived risk of contracting COVID-19 (called “infection risk” hereafter), perceived risk of becoming seriously ill from COVID-19 (“severity risk”), perceived risk of infection despite vaccination (“post-vaccination infection risk”), and perceived overall seriousness of the situation (“seriousness”). 

Attitudinal measures included confidence in the COVID-19 vaccines (“confidence”), belief in the importance of vaccination (“importance”), general support of vaccines (“support”), and past flu shot uptake (“past vaccination”). In addition, self-rated COVID-19 knowledge (“knowledge”) and intention to receive a booster (“booster intention”) were measured, all via Likert scales. Higher scores represented a higher inclination to vaccinate. “Prefer not to say” responses were coded as missing, and “Other” or “Unsure” responses were recoded or treated as missing as appropriate.

#### 2.2.3. Social Influence

To assess social influence, participants were asked about the perceived vaccination status of peers. A distinction was made between frequent contacts (i.e., classmates or coworkers) and friends to investigate whether vaccination decisions were shaped more by proximity or personal connection and trust.

### 2.3. Data Analysis

Correlation analyses examined associations between vaccination timing and predictors, followed by Kruskal–Wallis tests to assess differences across DOI groups. Significant differences were assessed using post hoc comparisons. Mediation analyses tested whether perceived COVID-19 seriousness and peers’ vaccination status influenced the effects of confidence and infection risk on speed to adoption (or vaccination timing). To extend beyond bivariate relationships and considering the nonlinearity of some of the relationships, a multinomial logistic regression (MNLR) was performed to examine predictor significance and the odds of each adoption group membership, designating laggards as the reference category. All theoretically relevant predictors were entered into the MNLR model to discover the relative influences of the indicators.

Predictors comprised categorical demographic variables (dummy-coded with reference groups: age ≥ 65, White, female) alongside continuous/ordinal covariates. Model assumptions were tested before interpretation, including independence of observations, mutual exclusivity and exhaustiveness of outcome categories, linearity in the logit for continuous predictors, absence of severe outliers or influential points, and multicollinearity evaluated using variance inflation factors (VIFs), with a conservative threshold of VIF < 3 indicating acceptable collinearity [48,49,50,51]. Significance was set at *p* < 0.05 for all analyses.

Analysis was performed via IBM SPSS Statistics (Version 31) [52] and the PROCESS macro extension (Version 4.3) [53] was used to conduct mediation analyses. Data visualization was conducted in Python (Version 3.14.3) using the Matplotlib library (Version 3.10.8) [54,55], with code developed in Visual Studio Code (Version 1.109).

## 3. Results

**Figure 1:**
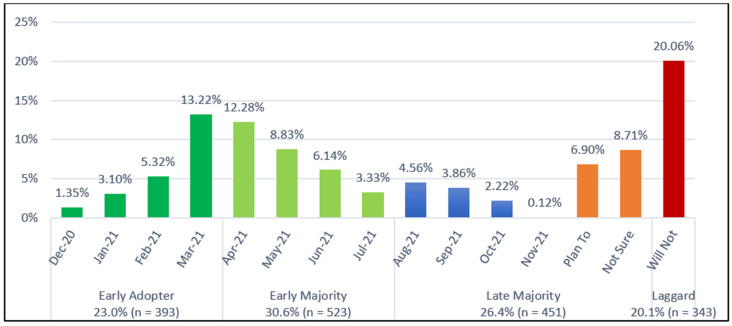
Timing of first COVID-19 vaccination and corresponding adoption groups in the U.S. (*N* = 1710). One year after vaccine rollout, 64.3% (1100) received at least one vaccine dose, and 35.7% (610; orange and red bars) were unvaccinated. Among the total sample, 20.1% (343; red bars only) expressed no intention to get vaccinated.

A total of 1710 individuals aged 16 years or older responded to the survey (participant characteristics presented in Table 1). Within one year of the COVID-19 vaccine rollout, 64.3% (*n* = 1100) had received at least one dose, with 1024 fully vaccinated. Among the 35.7% not yet vaccinated (610, presented in orange and red bars, Figure 1), 56.2% (343, or 20.1% of the sample) reported no intention to get vaccinated (red bar).

### 3.1. Demographic, Behavioral, and Attitudinal Factors

Vaccine adoption timing was significantly associated with multiple demographic and psychosocial factors (all *p* < 0.001). Among demographic variables, education exhibited the strongest positive correlation with earlier vaccination (r = 0.264), followed by income (r = 0.251) and age (r = 0.216), indicating that individuals who were older, had obtained more years of formal education, and were in the higher income groups (among the five levels presented in Table 1) tended to vaccinate earlier. Both past and future vaccination behavior were associated with earlier uptake, including the pattern of obtaining flu shots (r = 0.393) and booster intention (r = 0.234). On the other hand, self-reported COVID-19 knowledge showed a weak but statistically significant association (r = 0.093). Non-parametric analysis indicated that prior flu shot pattern differed across all four groups at each phase (H(3) = 310.82, *p* < 0.001). Knowledge also varied (H(3) = 13.69, *p* = 0.003) but the pattern was inconsistent; pairwise comparisons showed a significant difference only between early adopters and later adopter groups.

**Figure 2:**
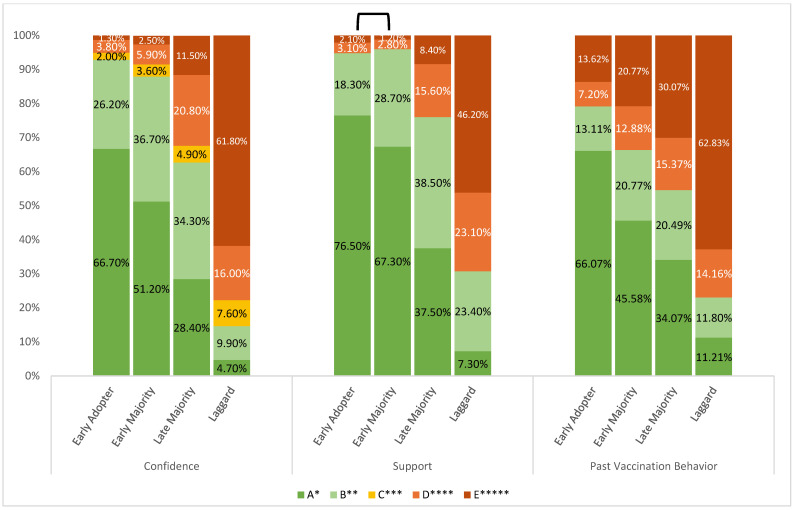
Portions of participants expressed levels of vaccine confidence, support, and past vaccination behavior across adoption groups (*n* = 1710). Ranges of response options (A to E) for confidence in the COVID-19 vaccine were as follows: very confident to very unconfident; general support of vaccines: strongly agree to strongly disagree; past-vaccination behavior (flu shot uptake): always or almost always to never. All three attitudes varied significantly across adoption groups (Kruskal–Wallis: H(3) = 671.79, 592.38, 310.82, respectively; all *p* < 0.001). The reversed U-shape on top of the bars indicates the pairs of groups that were insignificant from one another.

Relating to attitudes, belief in vaccine importance (r = 0.604; H(3) = 708.16, *p* < 0.001), vaccine confidence (r = 0.599; H(3) = 671.79, *p* < 0.001), and general support for vaccination (r = 0.562; H(3) = 592.38, *p* < 0.001) were associated with adoption speed. All showed variations across groups, with consistent pairwise differences except between early adopters and early majority (for importance and support). Later adopters, particularly laggards, reported more skepticism and lower perceived importance. Past vaccination behavior showed a clear gradient across adopter groups, with the proportion reporting “always or almost always” obtaining flu shots decreasing from 66.07% among early adopters to 11.21% among laggards (Figure 2).

### 3.2. Perceived Infection, Severity, Post-Vaccination, and Situational Risks

Likewise, perceived infection risk (r = 0.415), seriousness of the situation (r = 0.279), and illness severity risk (r = 0.150) were associated with earlier uptake. On the other hand, those who believed that post-vaccination risk was likely tended to delay or refuse the vaccine (r = 0.330).

**Figure 3:**
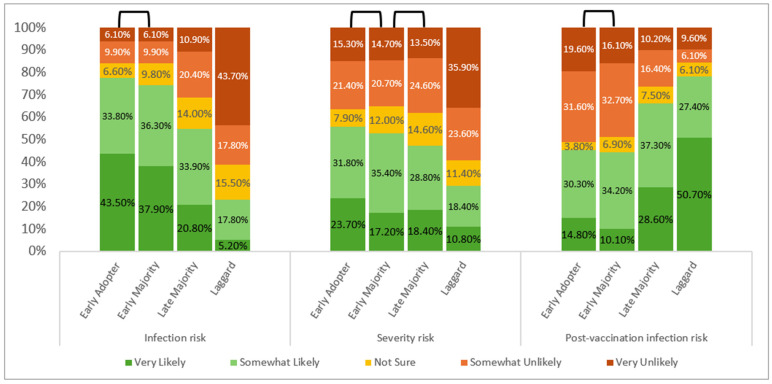
Levels of perceived COVID-19 infection risk, severe illness risk, and post-vaccination infection risk across adoption categories (*N* = 1710). Responses were 5-point Likert scales ranging from very likely to very unlikely. All three perceptions varied significantly across adoption groups (Kruskal–Wallis: H(3) = 353.73, 79.64, 204.04, respectively; all *p* < 0.001). The reversed U-shape on top of the bars indicates that the pairs of groups were insignificant from one another.

Significant differences were observed across the four adoption groups (all *p* < 0.001), yet each followed distinct patterns (Figure 3). Infection risk was highest among early adopters and declined across later groups, though pairwise comparisons showed that differences between the first two groups—early adopters and early majority—were insignificant. Severity risk showed little variation among the first three groups—early adopters, early majority, and late majority (*p* > 0.05); laggards deviated from them (*p* < 0.001), with 59.5% believing severe illness was somewhat/very unlikely. Post-vaccination infection risk revealed a reverse trend. Early adopters and early majority expressed stronger belief in vaccine protection, whereas over three-quarters of laggards believed infection after vaccination was still somewhat/very likely (Figure 3), citing it as a main reason for rejecting the vaccine.

### 3.3. Social Influence on Vaccination Speed

**Figure 4:**
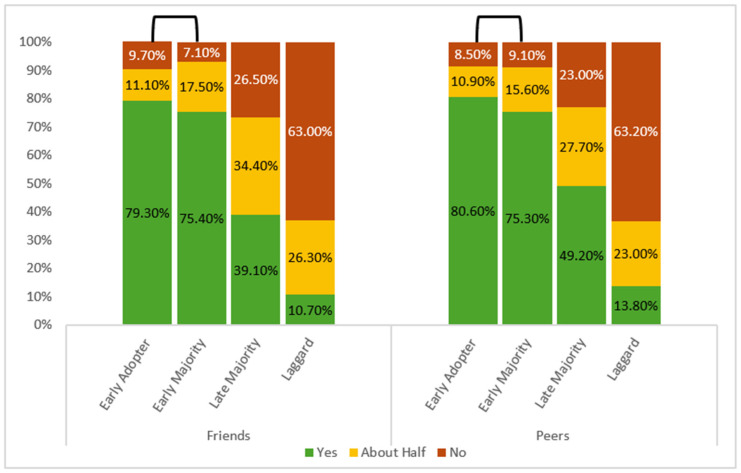
Perceived vaccination status of friends and peers across vaccine adoption groups (*N* = 1710). Participants indicated whether most friends and classmates/coworkers were vaccinated (Kruskal–Wallis: H(3) = 452.102 and 247.565, respectively; both *p* < 0.001). The reversed U-shape on top of the bars indicates the pairs of groups that were not significantly different from one another.

Both social influence factors were significantly associated with adoption speed, with individuals who had more friends (r = 0.542) or peers (r = 0.496) who were already vaccinated also obtaining vaccination earlier. Reported vaccination levels among friends and peers were similar between early adopters and early majority but differed significantly with later groups (friends: H(3) = 452.102; peers: H(3) = 247.565; both *p* < 0.001). The percentages declined steadily from early adopters to laggards, while non-vaccinated rates increased across later groups (Figure 4). At the same time, a substantial share of participants reported uncertainty regarding their peers’ vaccination status: 17.5% unsure about friends and 42.4% for coworkers or classmates. These responses were excluded from the primary analysis.

### 3.4. Mediation Analysis and Multinomial Regression

Vaccine confidence (β = 0.58, SE = 0.10, *p* < 0.001) and perceived infection risk (β = 0.29, SE = 0.09, *p* = 0.001) were clear indicators of adoption timing. In exploring potential mediating effects, peer vaccination status was found to partially mediate the relationship between confidence and adoption timing (β = 0.04, BootSE = 0.02, 95% CI [0.01, 0.09]) but not with infection risk. Perceived seriousness did not mediate either relationship.

**Figure 5:**
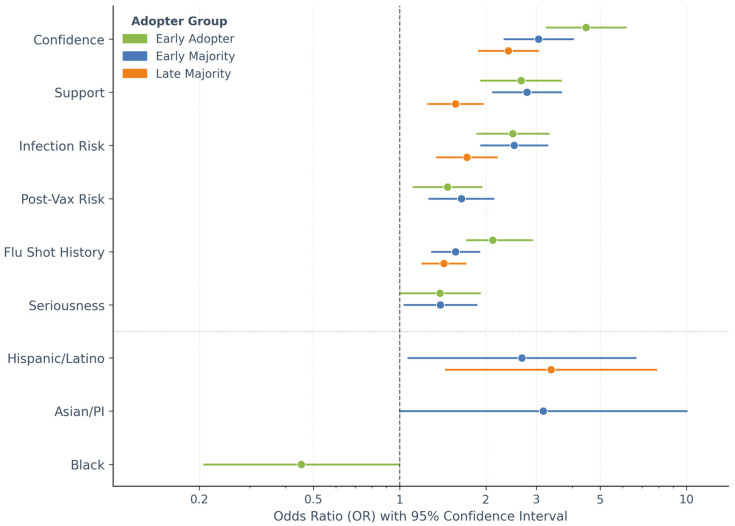
Significant predictors of vaccine adoption timing group membership. Odds ratios (ORs) from multinomial logistic regression are shown for statistically significant predictors (*p* < 0.05) across three adopter groups relative to the laggards group (reference category). Dots represent point estimates; respective accompanying horizontal lines indicate the 95% confidence intervals. The vertical dashed line at OR = 1 denotes no effect. Predictors below the dotted horizontal divider represent race/ethnicity variables; those above represent behavioral and attitudinal covariates.

MNLR results are presented in Table 2, and the odds ratios (OR) of significant indicators are plotted in a coefplot (Figure 5), with laggards as the reference category. All model assumptions were assessed and met, as described in Section 2. The model showed acceptable fit and strong explanatory power: Nagelkerke R^2^ = 0.602 (>0.4 indicates a strong relationship); likelihood-ratio test *p* < 0.001.

No significant gender differences were observed across groups. Compared with adults aged 65+, younger groups had lower odds of early adoption (all *p* < 0.05), while no significant age differences emerged for the two middle groups—early and late majorities. Black respondents were less likely to be in the initial group—early adopters (OR = 0.454, *p* = 0.048)—whereas Asian and Hispanic/Latino respondents had higher odds of being in the early or late majority groups.

Behavioral history and risk perceptions significantly differentiated adopter groups. Prior flu-shot history was associated with increased odds of being in the early adopter (OR = 2.111), early majority (OR = 1.567), and late majority group (OR = 1.425; all *p* < 0.001) relative to laggards. Higher perceived infection risk was similarly associated with increased odds of membership in all earlier adoption groups (all *p* < 0.001). Lower perceived post-vaccination infection risk, signaling stronger trust in the vaccine efficacy, was associated with higher odds of being an early adopter (OR = 1.468, *p* = 0.006) or early majority (OR = 1.640, *p* < 0.001). However, perceived severity was not significant across adopter groups.

Participants who reported greater pandemic seriousness were more likely to be an early adopter or early majority, but no significant differences were observed between the latter two groups. Those who expressed confidence in and support for the vaccine had very low odds of being laggards. Self-reported knowledge level was not associated with adopter group membership.

## 4. Discussion

This study examined vaccine behavior and perceptions by not only identifying *who* or *why* but also *how quickly* individuals would obtain vaccination. The temporal factor is critical in predicting and controlling the spread of disease. The pandemic provided a rare opportunity to investigate the progression of adopting a new vaccine at the population level, and the application of the DOI framework offered more nuanced insights into demographic, behavioral, and psychosocial factors. The findings provide additional evidence of the direct effects of confidence, risk perceptions, and social norms on adoption timing, as well as a mediating effect of peer vaccination status in the relationship between confidence and uptake.

Older adults were more likely to get vaccinated early, reflecting both their elevated COVID-19 infection risk and prioritization during the initial vaccine rollout—a strategy similarly adopted across countries globally and recommended by the World Health Organization (WHO) [56,57]. Due to the initial supply shortage [58] during the first few months in the U.S., frontline healthcare professionals and those who worked or lived in long-term care facilities were the first to obtain vaccination, followed by people aged 65 or older and front-line essential workers, stipulated by federal recommendation, with variation by states [59]. Such prioritization rendered higher vaccination rates among older adults worldwide (e.g., Germany and the UK observed 84% and 97% for adults over 65, respectively, compared to 78% in the U.S. [60]). Multinomial analyses confirmed that age exerted its strongest influence over the earliest phase, when limited availability and risk-focused messaging disproportionately favored older adults [61].

Higher education and income levels also predicted earlier acceptance, likely due to enhanced health literacy [39], trust in science [62,63], and better healthcare access [64,65]. Until spring 2021, COVID-19 vaccine demand had exceeded supply. Many individuals in the U.S., even when they became eligible according to their jurisdiction’s allocation plan, waited days or weeks for an appointment; the shortage was particularly severe and persisted in lower-resourced nations [66,67,68]. However, the relationship between education and trust in science is not universal; across 142 countries, the positive association was weaker or nonexistent in highly corrupt countries, suggesting that institutional context moderates the role of education in vaccine-related decision-making [69]. Once vaccines became widely available, these demographic factors were no longer strong determinants of uptake timing. Such diminishing influences were often masked in prior binary studies that did not consider the progression of adoption over time, demonstrating the benefit of applying the DOI framework.

The different dimensions of risk perceptions exhibited varied effects and were further distinguished here. Extending the existing literature [39,70], infection risk, along with several attitudinal factors, was found to be positively associated with speed to vaccination. Research on coping responses indicated that higher perceived threat increased willingness to be vaccinated [71]; a similar association was reported in Australia and Slovenia [72,73]. The observed increase in uptake during August and September 2021 followed the surge in confirmed cases in late summer [7]. Nevertheless, this effect could be attenuated by anticipated severity risk: if the chance of getting very sick was low, then the urgency of avoiding infection seemed trivial. Evidenced by the indifference observed among the first three groups that diverged from laggard, severity risk may persuade the hesitant to get vaccinated but be insufficient to motivate earlier adoption.

Moreover, belief in lower post-vaccination infection risk was positively associated with uptake decision and timing. It aligned with documented hesitancy regarding the COVID-19 vaccine, which primarily stems from safety and efficacy concerns, e.g., the technology was too new, the vaccine was developed too quickly, or the approval process was too rushed [33,39,74,75]. The results revealed that over three-quarters of laggards believed infection after vaccination was still somewhat or very likely. While this belief is not entirely wrong—since no vaccine is 100% effective at preventing infection [76,77]—it suggests that laggards may over-emphasize this limitation, contributing to a misconception that vaccines are ineffective and thus not worth taking.

On the other hand, self-rated COVID-19 knowledge was only weakly associated with vaccination timing and showed no consistent pattern across adopter groups. Even among those who reported being well-informed, vaccine decisions may hinge on trust, values, and perceived risks. Research using Protection Motivation Theory (PMT) found that in Taiwan, perceived knowledge enhanced coping appraisals—individuals’ positive perceptions of the COVID-19 vaccine as an effective prevention strategy—and led to uptake [78]. An American study of adolescents reported that perceived knowledge moderated the relationship between risk perception and vaccination among the low-knowledge group [79]. In the UK, however, knowledge was not predictive—likely due to misinformation and low institutional trust [80]. Consistent with this, MNLR did not identify self-rated knowledge as a significant predictor of earlier adoption, suggesting that information alone was insufficient to accelerate uptake. These findings underscore the need to communicate vaccine benefits more clearly to instill trust: they are intended to reduce hospitalization, morbidity, and mortality, not to completely eliminate infection [78]. Future studies could compare self-reported vs. objectively measured knowledge to help elucidate the discrepancy in the literature.

Attitudinal and behavioral factors, particularly belief in vaccine importance and confidence, consistently predicted earlier uptake, with confidence showing the clearest gradient across adopter groups. This mirrors findings from Australia, where use of trusted information sources such as government health websites and healthcare provider recommendations was among the strongest predictors of vaccine confidence and uptake [81]. Viewed through the DOI framework, these factors not only facilitated vaccination decisions but also could expedite adoption—key to disease prevention and epidemic control. In contrast, while perceived infection risk exerted a direct influence on uptake speed, severity did not independently predict adoption timing once confidence and infection risk were considered. Support for vaccines, including flu shot history, also contributed to earlier adoption, reflecting broader preventive health habits and compliance with public health recommendations among earlier groups. Understanding and applying the differing and interacting impacts of these facilitators are important, especially considering the continuously evolving SARS-CoV-2 virus and resurgence of several infectious diseases. Since the pandemic, the public has become vaccine-fatigued [82,83]. Messages and promotional strategies must be reevaluated to encourage adoption of subsequent boosters and timely vaccination.

While three in ten in the U.S. had not been vaccinated by the end of 2021, either still hesitated or refused (relatively high compared to 18.47% in Canada and 11.59% in China) [7], a notable subset of the population (20.1%) expressed no intention to get vaccinated, despite sufficient supply and continuously rising confirmed cases [84]. This rejection proportion remained stable through May 2023, when the last national cumulative data were reported, coinciding with the WHO’s declaration of the end of the pandemic [85]. Similarly, the unvaccinated ratio was 26.66% in Asia and 35.37% globally at the time and plateaued [7]. This group represents entrenched resistance rather than temporary reluctance, indicating that access and information alone may be insufficient to shift attitudes. Given this, it would not be productive to endeavor to change their minds, as success is unlikely. Instead, public efforts should focus on those who are hesitant but still willing to consider vaccination and motivate them to adopt the vaccine earlier or faster. This points to the need to examine the differences in group drivers to tailor approaches to specific populations.

Social influence has been identified as a strong promoter of behavior, including vaccination [86,87]. Early adopters and early majority were shown to report having a much higher proportion of their network vaccinated (75–80% responded most of their friends or peers had already been vaccinated) than later groups. Similarly, among UK adolescents, vaccination decisions were primarily shaped by parents, healthcare providers, and peers rather than social media, underscoring the cross-national relevance of social influence on vaccine behavior [88]. Conversely, being surrounded by unvaccinated people may shape reluctance or stimulate refusal. The pronounced disparity in peer vaccination between early and later groups suggests the operational function of social norms in the diffusion process. As vaccination became more common within close social networks, uncertainty may have diminished, enabling hesitant adopters to move toward vaccination. A study applying Social Cognitive Theory similarly identified observational learning—waiting to see whether friends and family get vaccinated first—as a significant predictor of vaccine behavior [89].

Mediation analyses further elucidate the dual roles of social influence in vaccination decision-making. People with higher vaccine confidence were more likely to perceive higher vaccination uptake among their peers, and these perceived norms were associated with earlier vaccination. This suggests that confidence acts both at the individual level and through perceived social norms, which in turn facilitate earlier vaccination.

The results also revealed that many people actively discussed COVID-19 vaccination with others, more frequently with friends than with broader peer groups. While perception can influence behavior, it may not be factually accurate. Earlier studies showed that individuals often misjudge peer behaviors, which public health campaigns have leveraged by correcting misperceptions and promoting positive social norms [86]. Others highlighted the role of subjective norms, particularly perceived approval from peers and family, in sustaining vaccine behaviors [90]. Messaging that emphasizes widespread uptake (e.g., “most students your age are vaccinated”) may help normalize vaccination and be particularly effective for younger, hesitant groups to move towards vaccination.

### Limitations and Future Research

This study has several limitations. First, data were collected via a self-administered survey, relying on participants’ recall and subjective perceptions, which may introduce bias or inaccuracies. For instance, knowledge was self-reported rather than objectively measured, which may explain part of the inconsistent effect on vaccine acceptance. Second, although actual uptake rather than intention alone was examined, the survey design cannot account for structural factors—such as access, mandates, or logistical barriers—that may have influenced vaccination behavior, especially during the early phase of rollout when there was a shortage. Finally, while the sample was relatively representative of the U.S. population and offered valuable insight into psychological and social drivers of vaccination timing, COVID-19 was a special case with rapid and widespread infection, high global mortality, daily news broadcasting, and a vaccine that employed a novel technology and was initially launched under emergency use authorization (EUA). These additional contextual factors may have intensified both the urgency to get vaccinated and, concurrently, hesitancy compared to other vaccines.

Future studies could examine the different influences between subjective and objective knowledge levels and their potential interaction effect. Similarly, given the strong role of social influence, it would be beneficial to further explore whether perceived norms and actual vaccination rate exert a similar impact on vaccination decision as well as timing.

## 5. Conclusions

Vaccine hesitancy studies have identified a range of factors influencing uptake decisions, primarily focusing on individual characteristics, including demographics, perceived risk, and attitudes (confidence in vaccine and authority or science, general support of such a preventive intervention). This study further distinguished various dimensions of risk perception, validated the influence of peer behavior, mapped actual vaccine adoption timing instead of hypothetical scenarios, and provided reference comparisons to other countries. In particular, the research examined these factors through the lens of the DOI framework, with the advantage of accounting for the progression of cumulated protection at the population level, expanding on and overcoming the deficiency of the more conventional dichotomized analysis of vaccinated vs. unvaccinated. Contextually, the pattern of uptake was also associated with availability, accessibility (both eligibility and ease of obtainment), and disease activity (levels of confirmed cases and mortality).

By identifying what separated early from late adopters, and what ultimately facilitated the hesitant to vaccinate, this study offers a more nuanced understanding of vaccination behavior and adds a critical temporal perspective that can inform and potentially improve future public health vaccination efforts. Using the DOI framework to focus on hesitant to vaccinate individuals and population groups using proven interventions to improve uptake, such as employing trusted community voices, clear provider communications, community-centered education and outreach, and the use of data to guide targeted outreach and vaccination programs has the potential to help individual providers, health departments, and health systems reverse the rising levels of vaccine hesitancy and declination of vaccination seen in the U.S. and globally [91]. Improving vaccine confidence and trust among those who are hesitant to vaccinate may facilitate more effective approaches to addressing other infectious diseases and mitigating public health threats, especially in our most vulnerable communities.

## Figures and Tables

**Table 1:** Participant self-reported demographic characteristics by adoption group, based on the timing of obtaining the first dose of COVID-19 vaccine (*N* = 1710).

	TOTAL % (*N*)	Early Adopters % (*n*)	Early Majority % (*n*)	Late Majority % (*n*)	Laggard % (*n*)
	100 (1710)	22.98 (393)	30.58 (523)	26.37 (451)	20.06 (343)
**Age Group**					
16–17	15.32 (262)	6.87 (18)	35.11 (92)	36.26 (95)	21.76 (57)
18–22	15.32 (262)	12.21 (32)	36.26 (95)	32.44 (85)	19.08 (50)
23–29	4.74 (81)	16.05 (13)	28.40 (23)	27.16 (22)	28.40 (23)
30–39	16.84 (288)	13.23 (52)	13.96 (73)	18.85 (85)	22.74 (78)
40–49	24.21 (414)	28.02 (116)	31.88 (132)	23.19 (96)	16.91 (70)
50–64	15.91 (272)	33.82 (92)	29.04 (79)	20.22 (55)	16.91 (46)
65+	7.66 (131)	53.44 (70)	22.14 (29)	9.92 (13)	14.50 (19)
**Gender**					
Male	46.55 (796)	28.52 (227)	31.16 (248)	22.49 (179)	17.84 (142)
Female	51.64 (883)	18.01 (159)	29.90 (264)	29.78 (263)	22.31 (197)
Other	1.81 (31)	22.58 (7)	35.49 (11)	29.03 (9)	12.90 (4)
**Race**					
Native American	1.64 (28)	10.71 (3)	17.86 (5)	35.71 (10)	35.71 (10)
Asian/Pacific Islander	5.73 (98)	17.35 (17)	53.06 (52)	20.41 (20)	9.18 (9)
Black/African American	12.40 (212)	9.91 (21)	26.89 (57)	41.04 (87)	22.17 (47)
Hispanic/Latino	8.36 (143)	17.48 (25)	35.66 (51)	36.36 (52)	10.49 (15)
White/Caucasian	68.77 (1176)	27.38 (322)	28.91 (340)	22.45 (264)	21.26 (250)
Other	3.10 (53)	9.44 (5)	33.96 (18)	33.96 (18)	22.64 (12)
Household Income *					
<$25,000	21.16 (251)	17.13 (43)	25.50 (64)	27.49 (69)	29.88 (75)
$25,000–$49,999	25.72 (305)	24.26 (74)	29.18 (89)	23.93 (73)	22.62 (69)
$50,000–$99,999	28.50 (338)	31.95 (108)	26.63 (90)	22.49 (76)	18.93 (64)
$100,000–$199,999	17.62 (209)	40.19 (84)	33.01 (69)	17.70 (37)	9.09 (19)
$200,000+	3.71 (44)	45.45 (20)	31.82 (14)	15.91 (7)	6.82 (3)
Prefer Not to Answer	3.29 (39)	35.90 (14)	25.64 (10)	23.08 (9)	15.38 (6)

* For reference, the median annual household income in the U.S. for 2024–2025 was $83,730. The average monthly cost of living is $2924 for a single person and $7101 for a family of four, with variation across states and cities.

**Table 2:** Predicting COVID-19 vaccine adoption timing groups via multinomial logistic regression (*N* = 1710).

Adopter Group ^a^	Parameter	B	Std. Error	Wald	df	Sig.	Odds Ratio (OR)	95% Confidence Interval for Exp(B)	
								Lower Bound	Upper Bound
**Early Adopter**	Intercept	−11.681	0.980	142.013	1	0.000			
	**Race (Factor Variable)**								
	Native American	−1.471	1.035	2.019	1	0.155	0.230	0.030	1.747
	Asian/PI	0.267	0.637	0.176	1	0.675	1.306	0.375	4.555
	Black	−0.789	0.399	3.917	1	0.048	0.454	0.208	0.992
	Hispanic/Latino	0.590	0.501	1.386	1	0.239	1.804	0.676	4.815
	White	0 ^b^			0				
	**Covariate Variables**								
	Flu shot history	0.747	0.105	50.527	1	0.000	2.111	1.718	2.594
	Infection risk	0.909	0.146	38.828	1	0.000	2.482	1.865	3.304
	Severity risk	−0.255	0.145	3.101	1	0.078	0.775	0.583	1.029
	Seriousness	0.325	0.162	4.016	1	0.045	1.384	1.007	1.902
	Confidence	1.496	0.161	85.886	1	0.000	4.465	3.254	6.127
	Support	0.975	0.163	35.657	1	0.000	2.651	1.925	3.651
	Knowledge	0.085	0.135	0.402	1	0.526	1.089	0.836	1.419
	Post-vac risk	0.384	0.139	7.608	1	0.006	1.468	1.117	1.928
**Early Majority**	Intercept	−10.385	0.913	129.483	1	0.000			
	**Race (Factor Variable)**								
	Native American	−0.695	0.831	0.700	1	0.403	0.499	0.098	2.544
	Asian/PI	1.153	0.587	3.857	1	0.050	3.168	1.002	10.015
	Black	0.013	0.332	0.001	1	0.970	1.013	0.529	1.940
	Hispanic/Latino	0.981	0.465	4.448	1	0.035	2.667	1.072	6.637
	White	0 ^b^			0				
	**Covariate Variables**								
	Flu shot history	0.449	0.097	21.646	1	0.000	1.567	1.297	1.894
	Infection risk	0.920	0.135	46.070	1	0.000	2.508	1.923	3.271
	Severity risk	−0.171	0.136	1.581	1	0.209	0.843	0.645	1.100
	Seriousness	0.327	0.147	4.921	1	0.027	1.387	1.039	1.851
	Confidence	1.117	0.140	63.453	1	0.000	3.054	2.321	4.020
	Support	1.022	0.140	53.134	1	0.000	2.778	2.111	3.656
	Knowledge	−0.050	0.124	0.162	1	0.687	0.951	0.746	1.213
	Post-vac risk	0.494	0.131	14.232	1	0.000	1.640	1.268	2.120
**Late Majority**	Intercept	−6.197	0.800	60.012	1	0.000			
	**Race (Factor Variable)**								
	Native American	0.189	0.659	0.082	1	0.774	1.208	0.332	4.395
	Asian/PI	0.255	0.587	0.189	1	0.664	1.290	0.409	4.075
	Black	0.498	0.290	2.939	1	0.086	1.645	0.931	2.905
	Hispanic/Latino	1.215	0.430	7.963	1	0.005	3.369	1.449	7.833
	White	0 ^b^			0				
	**Covariate Variables**								
	Flu shot history	0.354	0.089	15.998	1	0.000	1.425	1.198	1.695
	Infection risk	0.540	0.122	19.517	1	0.000	1.715	1.350	2.179
	Severity risk	0.056	0.124	0.208	1	0.649	1.058	0.830	1.348
	Seriousness	0.183	0.128	2.025	1	0.155	1.200	0.933	1.544
	Confidence	0.873	0.121	51.999	1	0.000	2.393	1.888	3.034
	Support	0.448	0.112	15.891	1	0.000	1.565	1.256	1.951
	Knowledge	−0.002	0.109	0.000	1	0.985	0.998	0.806	1.236
	Post-vac risk	0.091	0.120	0.579	1	0.447	1.096	0.866	1.387

a. The reference category is laggard. b. White participants were designated as the reference group; this parameter is set to zero because it is redundant.

## Data Availability

The data presented in this study are available on request from the corresponding author one year from the date of publication upon reasonable request with a methodologically sound research proposal.
